# 

**Published:** 1894-12

**Authors:** 


### CONTENTS.

ORIGINAL ARTICLES—

Plastic Operations in Carcinoma of the Breast. By J. T. Wilson, M. D., Sherman, Tex ........ 641
Some Causes That Lead to Invalidism in Women. By J. B. S. Holmes, M. D., Atlanta, Ga........ 647
Dengue. By Ira W. Porter, M. D., Sanford, Fla ........ 659
Transfusion in the Treatment of Acute Hemorrhages. By T. J. Crofford, M. D., Memphis, Tenn........ 662
Lecture on Extra-Uterine Pregnancy. By James A. Goggan, M. D., Alexander City, Ala........ 664

SOCIETY REPORTS—

Maryland Clinical Society........ 667

BOOK REVIEWS -

Text-Book of Hygiene. By G. H. Rohe, M. D ........ 673
The Pocket Anatomist. By C. Henri Leonard, A. M., M. D........ 675
The Physician's Visiting List for 1895........ 676

### CONTENTS.—Continued.

SELECTIONS AND ABSTRACTS—

The Tincture of Aconite for Tetanus .... 646
A Question of Ownership .... 658
Sprained Ankles .... 661
Formulæ for the Antiseptic Treatment of Boils and Carbuncles .... 672
A New Essay on Man .... 677
Labor and Heart Disease .... 676
Apocynum Cannabinum in Heart Disease .... 677
The Effect of Creosote on the Virulence of the TuberculiiBacillus 678
On the Reduction of Subcoracoid Discolation of the Humerus .... 679
Oxalic Acid in Combination With Iron and Mangnese Peptonates 680
Technique of Making Urethral Injections .... 680
The Advantages of Atmospheric Distension of the Rectum, With Dislodgement of Small Intestines .... 681
Infantile Therapeutics .... 682

EDITORIALS—

The After-Clap .... 684
Sir Joseph Lister .... 686
Editorial Notes .... 687–688
Prescriptions .... 690–691
Special Notes .... 692–696

